# Event-Based Prospective Memory Deficit in Children with ADHD: Underlying Cognitive Factors and Association with Symptoms

**DOI:** 10.3390/ijerph18115849

**Published:** 2021-05-29

**Authors:** Floriana Costanzo, Elisa Fucà, Deny Menghini, Antonella Rita Circelli, Giovanni Augusto Carlesimo, Alberto Costa, Stefano Vicari

**Affiliations:** 1 Child and Adolescent Psychiatry Unit, Department of Neuroscience, Bambino Gesù Children’s Hospital, IRCCS, 00146 Rome, Italy; elisa.fuca@opbg.net (E.F.); deny.menghini@opbg.net (D.M.); antonella.circelli@gmail.com (A.R.C.); stefano.vicari@opbg.net (S.V.); 2 Laboratory of Clinical and Behavioral Neurology, Santa Lucia Foundation, 00179 Rome, Italy; memolab@hsantalucia.it (G.A.C.); a.costa@hsantalucia.it (A.C.); 3 Department of Systems Medicine, Tor Vergata University, 00133 Rome, Italy; 4 Department of Psychology, Niccolò Cusano University, 00154 Rome, Italy; 5 Department of Life Sciences and Public Health, Catholic University, 00168 Rome, Italy

**Keywords:** ADHD, prospective memory, reward, attention

## Abstract

Event-based prospective memory (PM) was investigated in children with Attention deficit/hyperactivity disorder (ADHD), using a novel experimental procedure to evaluate the role of working memory (WM) load, attentional focus, and reward sensitivity. The study included 24 children with ADHD and 23 typically-developing controls. The experimental paradigm comprised one baseline condition (BC), only including an ongoing task, and four PM conditions, varying for targets: 1 Target (1T), 4 Targets (4T), Unfocal (UN), and Reward (RE). Children with ADHD were slower than controls on all PM tasks and less accurate on both ongoing and PM tasks on the 4T and UN conditions. Within the ADHD group, the accuracy in the RE condition did not differ from BC. A significant relationship between ADHD-related symptoms and reduced accuracy/higher speed in PM conditions (PM and ongoing trials), but not in BC, was detected. Our data provide insight on the adverse role of WM load and attentional focus and the positive influence of reward in the PM performance of children with ADHD. Moreover, the relation between PM and ADHD symptoms paves the road for PM as a promising neuropsychological marker for ADHD diagnosis and intervention.

## 1. Introduction

Attention deficit/hyperactivity disorder (ADHD) is a neurodevelopmental disorder characterized by a pattern of inattention and/or hyperactivity-impulsivity that interferes with functioning or development. Manifestations of the disorder must be present in more than one setting such as home and school [1].

ADHD occurs in about 5% of children and adolescents and about 2.5% of adults [2,3]. Besides the primary symptoms of inattention and/or hyperactivity, children and adolescents with ADHD exhibit impairment in a wider range of daily-life and academic domains. For instance, they show great difficulties in organizing and completing activities and in retaining information that they need in certain contexts (e.g., mental arithmetic, task instructions). Moreover, they often forget to perform daily activities, such as writing down homework, doing chores, running errands, returning a toy to a friend, bringing a jacket when going on a school trip [1,4,5].

### 1.1. Prospective Memory in ADHD

Some of the clinical manifestations of ADHD have been related to impairment in multiple cognitive domains involved in the complex sequence of behaviors required in prospective memory (PM) tasks. PM is the capability to remember to carry out future intentions—for instance, to remember to give someone a message at a certain time (time-based PM)—or event (event-based PM)—for instance, to remember to buy medicine next time you pass a pharmacy [6,7]. Importantly, the appropriate cue for carrying out the planned action is always embedded in ongoing activities (or ongoing tasks) interposed between intention formation and the critical moment of realization. During the execution of a task involving PM activation, the subject has to interrupt the ongoing task to initiate and perform the intended action at the appropriate moment. Mutual interaction between ongoing tasks and PM performances has been documented. Particularly demanding ongoing tasks negatively affect PM performances [8] and, furthermore, a “PM interference effect” [9,10] has been recognized, indicating a decrease in accuracy or speed in an ongoing task when a PM task occurs [11,12].

The neural basis of PM has been traced in the activity of the rostral prefrontal cortex [13,14] in association with other brain regions such as the anterior cingulate gyrus, the precuneus, and the temporal lobe [15,16,17,18]. The rostrolateral prefrontal cortex interacts with the putamen to maintain representations of future intentions [19], whereas the connectivity between the anterior prefrontal cortex and the precuneus has been reported to support the top-down sustained monitoring process [20].

PM is a critical issue in children with ADHD, as they often forget to execute their planned actions [21,22,23,24,25]. It has been suggested that PM mediates, at least partially, the relationship between the symptomatology of ADHD and procrastination behavior [26]. Compromised performances on PM tasks were also described in adults with ADHD, in association with a large-scale impairment in planning abilities; importantly, such difficulties have been confirmed not only in laboratory-based PM tasks, but also in naturalistic experimenter-assigned tasks [27,28]. In children with ADHD, the documented hypoactivation of key regions related to PM, in particular, the frontoparietal network [18], may play a role in PM deficits in this population.

Despite consistent findings of the presence of PM deficits in individuals with ADHD, investigations on a possible dissociation between deficits in time-based PM and event-based PM provided more contrasting results. A review by Talbot and Kerns [29] found that, although current literature agrees on the existence of time-based PM deficits in individuals with ADHD, there are inconsistent reports about the performances on event-based PM tasks in this population. Moreover, considering the possible influence of PM tasks on ongoing tasks, the evaluation of the impact of PM tasks on ongoing tasks could provide crucial insights to set up tailored interventions to improve overall functioning. Thus, further investigations on these topics are required.

### 1.2. Cognitive Factors Underlying Prospective Memory Performance

The cognitive mechanisms mediating performances on PM tasks in children with ADHD have not been extensively investigated. Literature suggests that factors such as working memory (WM) load and attentional focus can modulate PM performances in the general population or other groups of clinical samples. The executive framework of PM development [30] claims that WM supports cue retention and retrieval in PM tasks. Moreover, according to Guynn [11] and Smith [31], the allocation of WM and/or attentional resources is required to monitor the environment for the occurrence of the PM target. WM is required in PM tasks to actively sustain the intention while the individual is involved in the ongoing task; accordingly, WM has been found to be a predictor of PM performance in children and adults [23,32,33]. Thus, the manipulation of WM load in experimental paradigms should influence PM performance, as suggested by a number of studies [34,35]. A recent study conducted in a group of healthy adults found that in the time-based condition, but not in the event-based condition, prospective remembering was affected by the reduction of WM resources [36]. The role of WM—together with time perception abilities—in PM performance in ADHD has been investigated by Mioni and colleagues [37], but only for time-based PM. The authors found that the relationship between WM abilities, time perception, and time-based PM performance was evident in the control group but not in the ADHD group. However, to our knowledge, there are no studies investigating the role of WM load in event-based PM performances of children with ADHD.

Attention is another crucial factor in the performance of PM tasks. Considering the focus of attention as a possible intervening variable, we can distinguish between focal and non-focal PM targets, which differ in the extent to which the ongoing task guides attention towards the target. PM targets in the center of the attention do not require additional search or monitoring processes for detection [11]. It is conceivable that event-based PM targets arising in the periphery of attention might be less effective in causing retrieval of the action to perform compared to cues arising within the focus of attention. Hicks and colleagues [38] found that if event-based PM targets occur in parts of the environment that are already receiving a good degree of focal processing, then the probability of detection might not be influenced by changes in their features. By contrast, event-based PM targets occurring in the periphery of focal attention can be manipulated to direct attention toward a feature that will trigger recollection of the intention. To date, no data are available on the role of focus of attention in PM performance of children with ADHD.

Another element to take into account when studying PM performances of children with ADHD is the reward amount. Individuals with ADHD exhibit an alteration of reward mechanisms, resulting in an aberrant sensitivity to reinforcement [39]. These alterations have been linked to the concept of delay aversion, which indicates the preference for small immediate rewards over large, delayed rewards [40,41]. Thus, it is conceivable that the number of rewards has a role in modulating PM performances of children with ADHD: in particular, an increased amount of reward should positively affect their performances. However, this hypothesis has never been tested in PM tasks.

Ascertaining the presence of an event-based PM deficit and its cognitive characteristics in children with ADHD could also have important consequences for clinical work. According to the current version of the Diagnostic and Statistical Manual of Mental Disorders [1], the diagnosis of ADHD is a strictly clinical process based on the assessment of an individual’s behaviors and features; reliable diagnosis cannot be made using standardized instruments. Studies investigating the neuropsychological profile of individuals with ADHD failed to produce consistent results concerning their cognitive profile [42]. Moreover, it has been demonstrated that standardized instruments evaluating executive control are not predictors of ADHD symptoms and long-term outcomes in ADHD [43,44]. For this reason, investigating the presence of a correlation between performance on Prospective Trials and clinical evaluation of children with ADHD could identify new possible clinical markers for such neurodevelopmental conditions.

In summary, given the established deficits in PM of individuals with ADHD, the presence of specific event-based PM impairment in this population should be evaluated in order to better define the possible role of cognitive variables in event-based PM deficits in ADHD and its relation with symptomatology.

### 1.3. The Current Study

In the present study, we assessed the presence of deficits in event-based PM in children with ADHD. Moreover, to address the role of cognitive variables possibly influencing PM performance in children with ADHD, we tested different hypotheses with specific reference to WM load, type of attentional focus, and amount of reward, which are known to be involved in both PM tasks execution and ADHD manifestations, at least in part. At this aim, we developed a novel experimental procedure consisting of five different conditions: one baseline condition only including the ongoing task, and four PM conditions, including the ongoing task and concomitant PM tasks, varying for the WM load, the attentional focus, and the reward amount.

We expected that the manipulation of different cognitive factors intervening in the event-based PM performance would help in clarifying the inconsistent reports on event-based PM deficits in the ADHD population. In this view, we predicted that:

(a) Children and adolescents with ADHD would show reduced performance compared to controls in several event-based PM tasks, expressed as reduced accuracy and increased response times for both the ongoing and the PM conditions. We also predicted that their performance would be modulated by the cognitive factors manipulated in the PM tasks.

Considering the possible role of WM and attentional deficit in the ADHD population, we hypothesized the following:

(b) The increase of WM load (number of PM targets) would specifically affect the performance of children with ADHD by reducing the response accuracy and increasing the response times for both the ongoing and the PM task compared to the other PM conditions.
(c) The occurrence of the PM targets outside the central focus of attention would affect the performance of children with ADHD by reducing the response accuracy and increasing the response times for both the ongoing and the PM task compared to the other PM conditions.

Moreover, in light of the documented aberrant sensitivity to reinforcement typical of individuals with ADHD, we hypothesized that:

(d) The increase in reward amount would positively influence the PM performance of children with ADHD in comparison with the other PM conditions.

We also investigated the possible relation between PM performance and the presence of ADHD-related symptoms. This would represent an important achievement to support clinical evaluation. For this reason, event-based PM performance was related to the scores at an extensively used instrument for clinical and research purposes in ADHD. We hypothesized that:

(e) A negative relationship would emerge between PM performance and the ADHD symptoms.

## 2. Materials and Methods

### 2.1. Participants

The study included 47 participants, 24 children with ADHD–ADHD group (15 males and 9 females) and 23 typically developing children–CON group (16 males and 7 females), matched for chronological age (CA), intelligence quotient (IQ), and sex distribution. Table 1 describes the characteristics of participants. All participants were Caucasian, middle socioeconomic status children. The imbalance between males and females mirrors the distribution of ADHD, which is more frequent in males than in females with a ratio of approximately 2:1 [1].

Selection criteria included, in addition to clinical diagnosis of ADHD, age between 8 and 13 years, and performances in the normal range on standardized intelligence tests. The age range was defined based on literature indicating that PM ability has been found to be acquired as early as 2 years of age [45] and to increase significantly around 7 and 8 years of age [46,47]. Exclusion criteria included reports of neurological signs and patients undergoing pharmacological treatments, as well as a history of language delay or learning disability.

Participants with ADHD were recruited at the Child and Adolescent Neuropsychiatry Unit of the Bambino Gesù Children’s Hospital in Rome; all of them were children undergoing the visit for the first diagnosis of ADHD. All participants underwent a child psychiatric and neuropsychological examination conducted by experienced developmental psychiatrists and neuropsychologists. The diagnosis was performed with the support of The Schedule for Affective Disorders and Schizophrenia for School-Age Children-Present and Lifetime Version (K-SADS PL), a clinical interview [48], and the administration of the standardized scale Conners’ Parent Rating Scales Long Version, Revised (CPRS) [49]. IQ was assessed using the Raven Coloured Progressive Matrices for children aged up to 11 years or the Raven Standard Progressive Matrices for children aged over 11 years [50]. Considering the clinical evaluation, 17 children out of 24 had a diagnosis of ADHD-combined presentation, and the other 7 children had a diagnosis of ADHD-predominant inattentive presentation. Of the participants with ADHD, 66% had at least one comorbid learning disorder. Controls were recruited at a primary and secondary public school in Rome and underwent the same IQ and CPRS-R scale evaluation. ADHD and CON groups did not differ for chronological age [F(1,45) = 0.44, *p* = 0.51], IQ [F(1,45) = 2.31, *p* = 0.14], and sex distribution [χ2 = 0.26, *p* = 0.61], but did differ for ADHD symptoms in the CPRS scale, particularly the CPRS total score and Oppositional, Cognitive problems/inattention, Hyperactivity, ADHD Index, Global index-Restless-impulsive, Global index, DSM Inattentive, and DSM hyperactive/impulsive CPRS-R subscales (all *p* < 0.001).

The experimental sessions were carried out after informed consent had been obtained from all participants and their families, and the study had been approved by the local Ethics Committee (process number 1111_OPBG_2016).

### 2.2. Study Design and Procedure

The PM assessment was administered on two different days. All participants were tested individually in a quiet, well-lit room. Participants received specific instructions for the execution of the experimental tasks and, before starting the test phase, they were asked to repeat the instructions to assure their comprehension. Each task lasted approximately 7 min; the overall experimental session lasted approximately 45 min.

#### 2.2.1. Assessment

The Raven’s Standard Progressive Matrices [50] is a 60-item test used to assess mental ability associated with abstract reasoning, considered a nonverbal estimate of fluid intelligence. The test consists of increasingly difficult pattern matching tasks and has little dependency on language abilities.

Conners’ Parent Rating Scales-Long Version, Revised (CPRS) [49] are widely used instruments for diagnostic and research purposes in the ADHD field, which can be administered to both parents and teachers. They assess core symptoms as well as symptoms of other behavioral and emotional disorders commonly associated with ADHD (e.g., oppositional behavior), based on DSM-IV-TR criteria [51]. CPRS is a multidimensional questionnaire; items are scored on a four-point Likert scale and divided into two subscales that assess symptoms related to ADHD: restless-impulsive and emotional lability. The scale has a cut-off for elevated (T 65-69) and for very elevated scores (T ≥ 70), indicating borderline and clinical scores, respectively. The questionnaire compiles scores in each of the following areas: Oppositional/defiant; Cognitive problems/inattention, Hyperactivity, Anxiety, Perfectionism, Social problems, Psychosomatic, ADHD index, Global index- Restless-impulsive, Global index-Emotional lability, Global index, DSM Inattentive, DSM hyperactive/impulsive, DSM Total.

The K-SADS PL is a clinician-administered semi-structured interview used for diagnosing children and adolescents aged 6 to 18 years according to DSM-IV criteria [48]. The screening interview (including the psychosis screen) is administered first; if threshold symptoms are detected during the screen, in-depth supplements for each category of diagnosis (e.g., psychotic disorders) are administered. It assesses both current and past psychopathology.

#### 2.2.2. Experimental Paradigm

The experimental paradigm consisted of one baseline condition (BC) including only the ongoing task, and four PM conditions, including the ongoing task and concomitant PM tasks. Stimuli were similar in visual characteristics but the task to be performed changed.

The ongoing task required the participants to make a semantic decision, pressing two different buttons of a PC keyboard in response to the stimulus appearing at the center of the screen. The PM task required participants to press another key (the space bar) when the PM target appeared. The PM targets, namely the event that marked the prospective task, varied between conditions. In one PM condition, the reward obtained for responses differed from the other conditions.

The four PM conditions were: 1 Target (1T), 4 Targets (4T), Unfocal (UN), and Reward (RE). 1T included only one PM target, presented at the center of the attentional focus (i.e., center of the screen, in correspondence of the fixation point), without reward manipulation; 4T included four different PM targets, at the center of the attentional focus, without reward manipulation; UN included one PM target, outside the center of the attentional focus (i.e., in correspondence of one of the four corners of the screen), without reward manipulation; RE included only one PM target, at the center of the attentional focus, but a reward manipulation occurred. Performance accuracy (proportion of correct responses) and response reaction time (RTs, milliseconds) to the ongoing trials and the PM trials were recorded.

##### Stimuli and Procedure

The participants were seated in a comfortable chair facing a 15” PC screen on a table about 40 cm away from them. E-Prime 2.0 software (Psychology Software Tools, Sharpsburg, PA, USA) was used to program and run the experiment and to record participants’ answers. Each PM condition included 108 ongoing task trials and 12 PM trials, for a total of 120 trials, instead, the BC included only 120 ongoing task trials. The PM target did not appear in the first four trials of a PM condition. Each trial started with a 200 ms fixation point, consisting of a black cross, 12 points Times New Roman, at the center of the screen, followed by the presentation of a task stimulus, appearing for 2 s. Response RTs and accuracy were recorded during the 2-s stimulus presentation. If a correct response was given, a reward stimulus appeared for 1 s immediately after the response; if incorrect or no response was recorded a different reward stimulus (a red cross indicating no reward) appeared.

Stimuli consisted of colored animal images (belonging to two categories: flying, non-flying animals), mean size 8 × 10 cm, located at the center of the screen, 3° × 3° to the fixation point; and four colored plant images, size 5 × 5 cm, located at the four corners of the screen, respectively 45°, 90°, 180°, 360° to the fixation point, in a white background, for a total of 600 images. Per each condition the stimuli varied: a list of 10 animal images (5 flying and 5 non-flying animals) was presented 12 times in the BC, 1T, UN, and RE conditions, while a list of 40 animal images (20 flying and 20 non-flying animals) was presented 3 times in the 4T condition (total of 90 different animal images). When the animal image was repeated, the surrounding set of plant images changed per each presentation. The sets of 4 plant images at the corners were arranged from a pool of 80 different plant images. A list of 40 plant images was chosen per each condition to create 120 combinations of 4 plant images at the corners, with each plant image presented 12 times. Specifically, 50 different new combinations were created, while 70 other combinations were created with a plant image previously associated with other plant images but in different positions. In this way, each plant image was presented associated with the same plant images, but in different positions, only two times (10 combinations), while for the other two times it was presented associated with 2 of the previous plant images. Thus, each animal image was associated with different plant image combinations. The reward stimuli consisted of the image of a yellow star above a bag, with the writing of the score obtained and the count of the points gained up to that moment. In the case of an error, the bag was covered with a red cross.

Each condition was preceded by a learning session, in which participants were given written task instructions and were shown examples of the stimuli in a PowerPoint presentation. For the PM conditions, the PM targets were previously presented in the PowerPoint presentation to be studied. A recognition test followed to be sure that the participant correctly recognized all the PM targets among distractors (different stimuli list from the experimental conditions).

Each experimental condition started after a short familiarization session of a computerized presentation of 10 trials.

The experiment started with the BC condition, and then the four PM conditions were administered in a counterbalanced order between participants.

##### Baseline Condition

**Figure 1:**
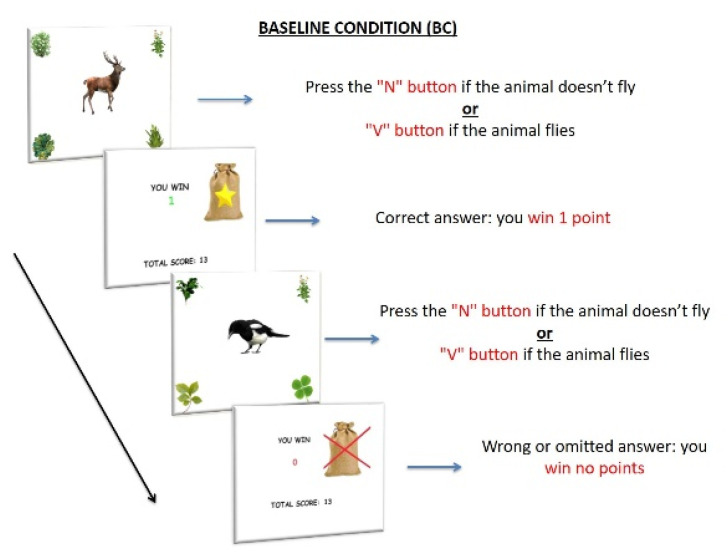
Procedure of Baseline condition (BC). The items consisted of 108 images of an animal located at the center of the screen; images of plants were located at the four corners of the screen. Participants had to press the “N” button when the image of a non-flying animal appeared on the screen and the “V” button when the image of a flying animal appeared on the screen (semantic decision task). Each stimulus appeared for 2 s. Participants received 1 RP for every correct answer and no RP for any wrong or omitted answer.

BC only included the ongoing task, for a total of 108 trials randomly presented. Participants were asked to perform a semantic decision task, pressing the “V” button, if an image of a flying animal appeared, and the “N” button if an image of a non-flying animal appeared (Figure 1). Participants received 1 reward point (RP) for every correct answer and no RP for any wrong or omitted answer.

##### PM Conditions

In the PM conditions, participants had to execute an event-based PM task (12 trials) while performing the ongoing task (108 trials). Specifically, the ongoing task must be interrupted to be able to carry out the PM task, when the PM target appeared on the screen. 

**Figure 2:**
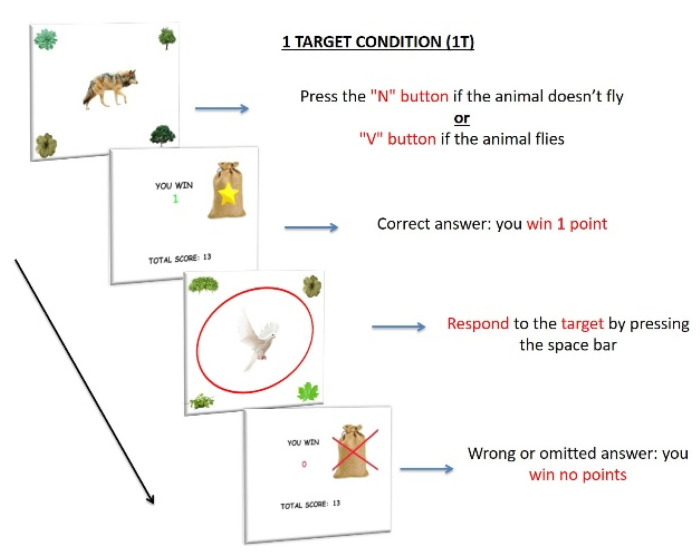
Procedure of 1 Target condition (1T). The items consisted of 120 images of an animal located at the center of the screen; images of plants were located at the four corners of the screen. As in the BC, participants had to press the “N” button when the image of a non-flying animal appeared on the screen and the “V” button when the image of a flying animal appeared on the screen (ongoing task). However, when a dove—PM target—appeared on the center of the screen (12 trials), participants were required to press the spacebar (PM task), instead of the semantic decision task. Each stimulus appeared for 2 s. They received 1 RP for every correct answer and no RP for any wrong or omitted answer, in both tasks (ongoing and PM trials).

1T. Only one PM target was included, at the center of the attentional focus, without reward manipulation. For the ongoing task, participants had to press the “N” button when the image of a non-flying animal appeared on the screen and the “V” button when the image of a flying animal appeared on the screen (ongoing task). However, when a dove—the PM target—appeared at the center of the screen (12 PM trials), participants were required to press the spacebar (PM task), instead of the semantic decision task (Figure 2). They received 1 response point (RP) for every correct answer and no RP for any wrong or omitted answer, in both trials (ongoing and PM trials).

**Figure 3:**
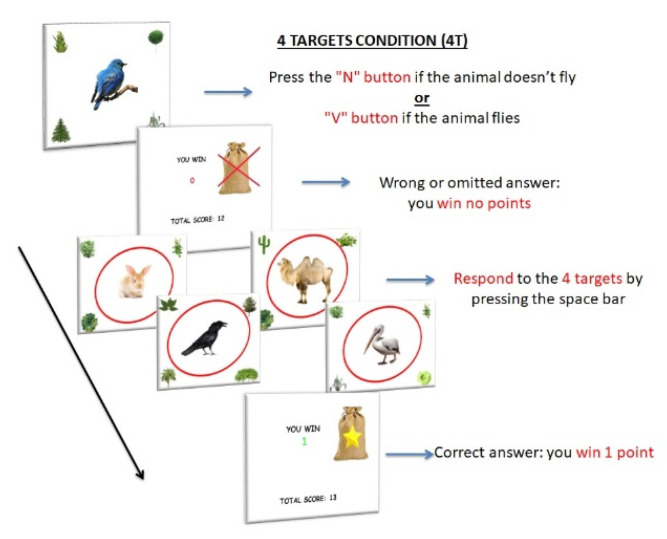
Procedure of 4 Targets condition (4T). The items consisted of 120 images of an animal located at the center of the screen; images of plants were located at the four corners of the screen. As in the BC, participants had to press the “N” button when the image of a non-flying animal appeared on the screen and the “V” button when the image of a flying animal appeared on the screen (ongoing task). However, when one of the four PM targets (rabbit, crow, camel, pelican) appeared at the center of the screen (12 trials), participants were required to press the spacebar (PM task), instead of the semantic decision task. Each stimulus appeared for 2 s. They received 1 RP for every correct answer and no RP for any wrong or omitted answer, in both tasks (ongoing and PM trials).

4T. Four PM targets were included, each one presented at the center of the attentional focus, without reward manipulation. For the ongoing task, participants had to press the “N” button when the image of a non-flying animal appeared on the screen and the “V” button when the image of a flying animal appeared (ongoing task). However, when one of the four PM targets (rabbit, crow, camel, pelican) appeared at the center of the screen (12 PM trials), participants were required to press the spacebar (PM task), instead of the semantic decision task (Figure 3). They received 1 RP for every correct answer and no RP for any wrong or omitted answer, in both trials (ongoing and PM trials).

**Figure 4:**
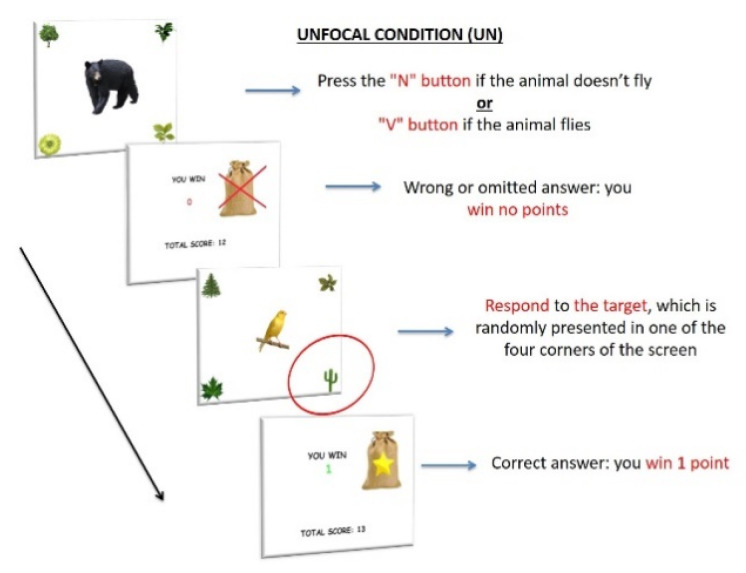
Procedure of Unfocal condition (UN). The items consisted of 120 images of an animal located at the center of the screen; images of plants were located at the four corners of the screen. As in the BC, participants had to press the “N” button when the image of a non-flying animal appeared on the screen and the “V” button when the image of a flying animal appeared on the center of the screen (ongoing task). However, when the cactus plant—Prospective target—appeared in one of the four corners of the screen (12 trials), participants were required to press the spacebar (PM task), instead of the semantic decision task. Each stimulus appeared for 2 s. They received 1 RP for every correct answer and no RP for any wrong or omitted answer, in both tasks (ongoing and PM trials).

UN. Only one PM target was included, outside the center of the attentional focus (i.e., in correspondence of one of the four corners of the screen), without reward manipulation. For the ongoing task, participants had to press the “N” button when the image of a non-flying animal appeared and the “V” button when the image of a flying animal appeared on the screen (ongoing task), at the center of the attentional focus (i.e., in correspondence of the fixation point). However, when the cactus plant—PM target—appeared in one of the four corners of the screen (12 PM trials), participants were required to press the spacebar (PM task), instead of the semantic decision task (Figure 4). They received 1 RP for every correct answer and no RP for any wrong or omitted answer, in both trials (ongoing and PM trials).

**Figure 5:**
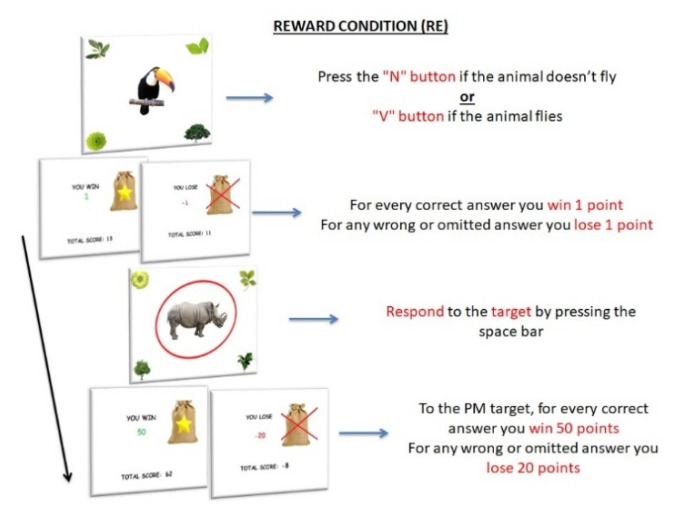
Procedure of Reward condition (RE). The items consisted of 120 images of an animal located at the center of the screen; images of plants were located at the four corners of the screen. As in the BC, participants had to press the “N” button when the image of a non-flying animal appeared on the screen and the “V” button when the image of a flying animal appeared on the screen (ongoing task). However, when a rhinoceros—PM target—appeared at the center of the screen (12 trials), participants were required to press the spacebar (PM task), instead of the semantic decision Table 2 seconds. In the ongoing task, they received 1 RP for every correct answer and lost 1 RP for any wrong or omitted answer. In the PM task, they received 50 RP for every correct PM answer and lost 20 RP for any wrong or omitted PM answer.

RE. Only one PM target was included, at the center of the attentional focus, but a reward manipulation occurred. In the ongoing task, participants had to press the “N” button when the image of a non-flying animal appeared on the screen and the “V” button when the image of a flying animal appeared on the screen (108 ongoing task trials). However, when a rhinoceros—PM target—appeared at the center of the screen, participants were required to press the spacebar (PM task), instead of the semantic decision task (Figure 5). Differently for the other PM conditions, in the ongoing task trials, they received 1 RP for every correct answer but lost 1 RP for any wrong or omitted answer. Moreover, in the PM trials, they received 50 RP for every correct answer and lost 20 RP for any wrong or omitted answer.

The comparison between the different conditions allowed us to evaluate the contribution of different cognitive variables, with specific reference to the WM load, the type of attentional focus, and the amount of reward, in the PM execution. In particular, 4T differed from 1T only for the number of PM targets, allowing us to evaluate whether the WM load affected event-based PM performance. UN condition differed from 1T only for the location of the PM target (center or corner of the screen), allowing us to evaluate whether the displacement of the PM target out of the attentional focus affected the event-based PM performance. RE condition differed from 1T only for the RP associated with both the ongoing and PM tasks, allowing us to evaluate whether possible changes in motivation, linked to the reward amount, could influence the PM performance.

### 2.3. Statistical Analyses

The demographic variables were compared through One-way Anovas. A chi-squared test was used to determine the non-parametric variables. To evaluate the effect of the conditions, mixed-design ANOVAs were performed on the mean proportion of accuracy and the mean response RTs separately for the ongoing task trials and the PM trials. For the ongoing task, Group (CON vs. ADHD) was considered as a between-subjects factor and Condition (BC vs. 1T vs. 4T vs. UN vs. RE) as a within-subjects factor; for the PM trials, Group (CON vs. ADHD) was considered as a between-subjects factor and Condition (1T vs. 4T vs. UN vs. RE) as a within-subjects factor.

Mauchly’s test was used to check for a violation of sphericity. The overall level of significance was set at *p* < 0.05. If a violation of sphericity was detected, a Greenhouse–Geisser correction was applied. Post hoc analyses were performed using Bonferroni’s test. Partial eta squared (ηp^2^) was used to measure effect size. The Pearson correlation was used to test the association between the experimental variables (accuracy and RTs of the ongoing and PM trials of each experimental condition) and the scores obtained on the CPRS global indexes in all groups. A Bonferroni correction for multiple comparisons was applied and, according to the number of comparisons, a different *p*-value was considered statistically significant (*p* < 0.0004).

## 3. Results

### 3.1. PM Procedure

#### 3.1.1. Ongoing Task—Accuracy

**Figure 6:**
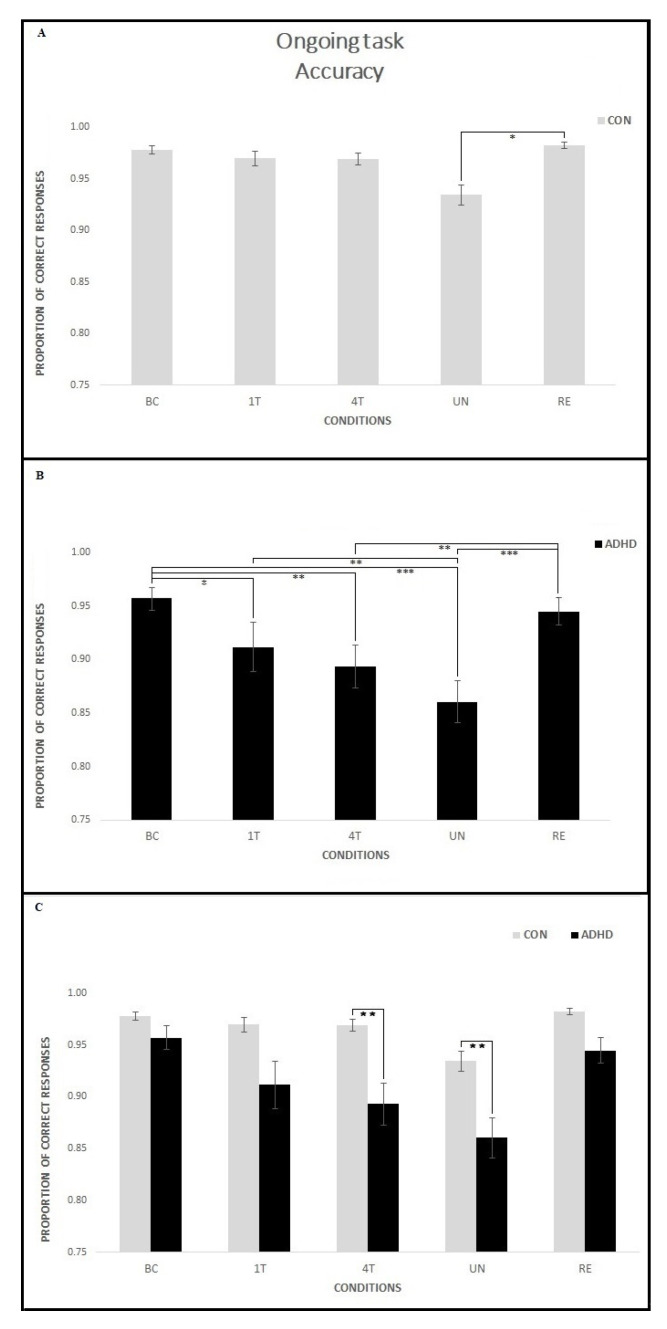
Accuracy on the ongoing task for each condition in the two groups. Within-group comparisons showed that in the CON group (**A**) no significant difference emerged between BC and all the PM conditions; the accuracy on the UN was lower than the RE condition. Conversely, participants in the ADHD group (**B**) showed lower accuracy in almost all the PM conditions compared to BC. 4T and UN conditions were lower than 1T and RE. Between-group comparisons (**C**) showed that the ADHD group performed significantly lower than the CON group on the 4T and UN conditions, while no difference emerged on the BC, 1T, and RE conditions. Asterisks mark significant differences: * *p* < 0.05, ** *p* < 0.01, *** *p* < 0.001.

Results showed a significant interaction Group per Condition [F(4,180) = 3.15; *p* = 0.02, *p* = 0.03 with Greenhouse–Geisser correction, *ηp*^2^ = 0.07]. Post hoc analysis revealed that performance of the ADHD group was significantly lower than that of the CON group on the 4T (*p* < 0.01) and UN (*p* < 0.01) conditions, while no difference emerged on the BC, 1T and RE conditions (all *p* > 0.1). Moreover, within-group comparisons showed that participants in the ADHD group had lower accuracy in almost all the PM conditions compared to BC (1T, *p* = 0.04; 4T, *p* < 0.01; UN, *p* < 0.001) with the exception of the RE, to which BC did not differ (*p* = 1.00). In addition, the accuracy on the UN was lower than 1T (*p* < 0.01) and RE (*p* < 0.001) conditions, and the accuracy on the 4T was lower than RE (*p* < 0.01). Conversely, within-group comparisons in the CON group showed no significant differences in accuracy between BC and all PM conditions (all *p* > 0.1). The accuracy on the UN was significantly lower than on the RE (*p* = 0.02). A general Group effect also emerged [F(1, 45) = 12.45; *p* < 0.001, *ηp*^2^ = 0.22] with the ADHD group showing lower accuracy (mean proportion of accuracy 0.91) than the CON group (mean proportion of accuracy 0.97). Finally, a general Condition effect emerged [F(4,180) = 17.7; *p* < 0.001, *p* < 0.001 with Greenhouse–Geisser correction, *ηp*^2^ = 0.28] because all groups showed the lowest accuracy in the UN compared to all other conditions (all *p* < 0.01); moreover ongoing task accuracy on the BC was higher than 1T (*p* < 0.05), 4T (*p* < 0.01) and UN (*p* < 0.001), but not than RE (*p* = 1.00). Figure 6 shows the mean proportion of accuracy in the ongoing task for each condition in the two groups.

#### 3.1.2. Ongoing Task—Speed

**Figure 7:**
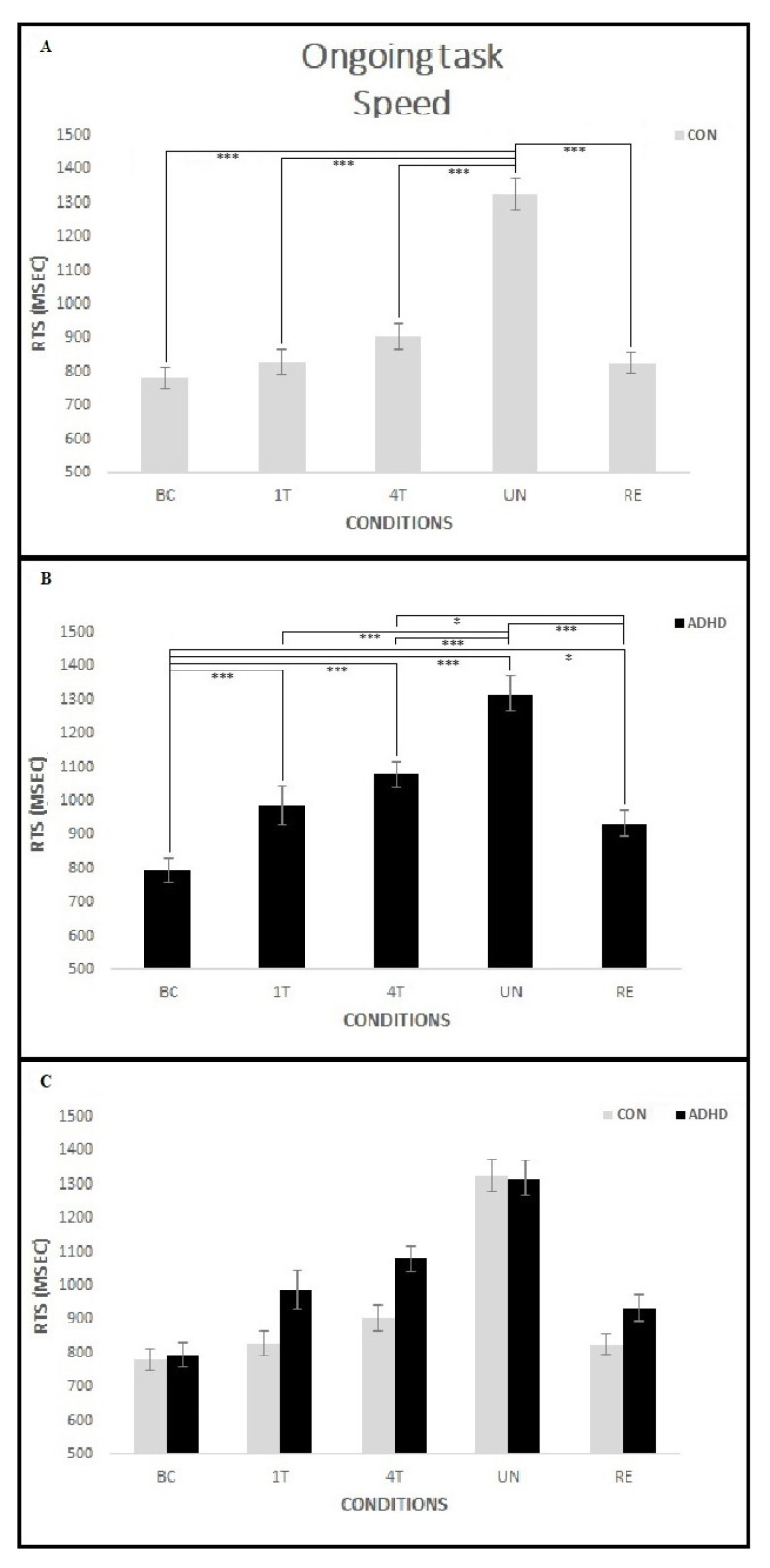
Ongoing task speed for each condition in the two groups. Within-groups differences emerged in the mean response RTs (see **A**,**B**), but no significant differences occurred between groups (**C**). In the CON group (**A**), no difference emerged between the mean response RTs in the BC and almost all PM conditions (1T, 4T, RE), with the exception of the UN, in which they were slower than all other conditions. In the ADHD group (**B**), participants were slower in all PM conditions compared to BC; moreover, as in the CON group, on the UN they were slower than all other conditions. Finally, the mean response RTs on the 4T was slower than the RE condition. Asterisks mark significant differences: * *p* < 0.05, *** *p* < 0.001.

The results showed a significant interaction Group per Condition [F(4,180) = 4.18; *p* < 0.01, *p* < 0.01 with Greenhouse–Geisser correction, *ηp*^2^ = 0.08]. However, post hoc analysis showed no significant differences in the mean response RTs between groups for all conditions (all *p* > 0.1), only within-group differences emerged. In particular, participants in the ADHD group were slower in all PM conditions compared to BC (all *p* < 0.05). Furthermore, on the UN, they were slower than all other conditions (all *p* < 0.001) and on the 4T, they were slower than the RE condition (*p* = 0.02). Conversely, in the CON group, no difference emerged between mean response RTs in almost all PM conditions (1T, 4T, RE) and BC (all *p* > 0.1), except for the UN, in which participants were slower than all other conditions (all *p* < 0.001). A general Group effect did not reach significance [F(1, 45) = 3.73; *p* = 0.06, *ηp*^2^ = 0.08] although the ADHD group showed a tendency to be slower (mean response RTs 1021.7 msec) than the CON group (mean response RTs 933.0 msec). Finally, a general Condition effect emerged [F(4, 180) = 100.86; *p* < 0.001, *p* < 0.001 with Greenhouse–Geisser correction, ηp2 = 0.69] because mean response RTs differed in each condition as follows: all groups were slower in the UN (mean response RTs 1320.6 msec) than in the 4T (mean response RTs 991.7 msec) and 1T (mean response RTs 907.9 msec) conditions (all *p* < 0.05), no difference emerged between the 1T and RE (mean response RTs 878.6 msec) conditions (*p* > 0.1), and all groups were slower in RE than BC (mean response RTs 787.8 msec), *p* = 0.02. Figure 7 shows the mean response RTs on the ongoing task for each condition in the two groups.

#### 3.1.3. PM Task—Accuracy

Results showed a significant Group effect [F(1, 45) = 10.59; *p* < 0.01, *ηp*^2^ = 0.19] with the ADHD group showing lower accuracy (mean proportion of accuracy 0.85) than CON group (mean proportion of accuracy 0.93). A general Condition effect also emerged [F (3,135) = 21.78; *p* < 0.001, *p* < 0.001 with Greenhouse–Geisser correction, *ηp*^2^ = 0.33] because all groups showed the lowest accuracy in the UN (mean proportion of accuracy 0.80) and 4T (mean proportion of accuracy 0.85) compared to the other conditions, (all *p* < 0.01—1T mean proportion of accuracy 0.93, RE mean proportion of accuracy 0.93). Moreover, 1T did not differ from 4T (*p* = 0.14).

Finally, the interaction Group per Condition was significant [F (3,135) = 3.40; *p* = 0.02, *p* = 0.04 with Greenhouse–Geisser correction, *ηp*^2^ = 0.07]. Post hoc analysis revealed that the ADHD group performed significantly lower than the CON group in the 4T (*p* = 0.02) and UN (*p* = 0.01) conditions, while no difference emerged in the 1T and RE conditions (all *p* = 1.0). Moreover, within-group comparisons showed that participants in the ADHD group showed lower accuracy in the 4T and UN compared to 1T (respectively *p* < 0.01 and *p* < 0.001) and RE (all *p* < 0.001) conditions. Conversely, within-group comparisons in the CON group showed no significant differences between conditions (all *p* > 0.1) except for the UN being lower than RE (*p* = 0.02).

**Figure 8:**
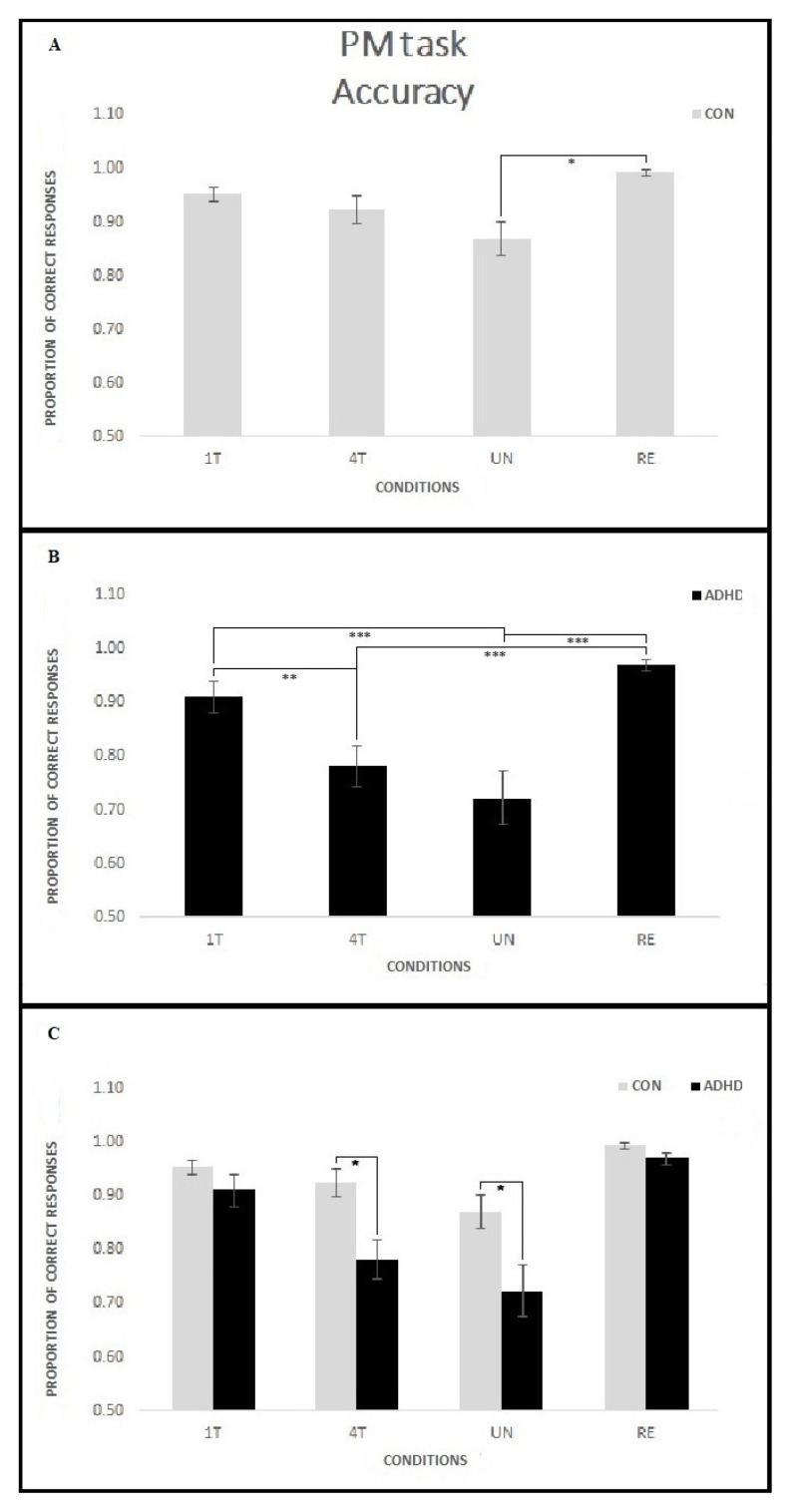
Accuracy on the PM trials for each PM condition in the two groups. Participants in the CON group showed no significant differences between conditions, with the exception of the UN, lower than the RE condition (**A**). Conversely, participants in the ADHD group showed lower accuracy on the 4T and UN conditions compared to 1T and RE (**B**). Moreover, differences between groups emerged because the ADHD group performed significantly lower than the CON group on the 4T and UN conditions (**C**). Asterisks mark significant differences: * *p* < 0.05, ** *p* < 0.01, *** *p* < 0.001.

Figure 8 shows the mean proportion of response accuracy on the PM task for each condition in the two groups.

#### 3.1.4. PM Task—Speed

The results showed a significant Group effect [F(1, 45) = 5.71; *p* = 0.02, *ηp*^2^ = 0.11] with the ADHD group being generally slower (mean response RTs 1126.1 msec) than CON group (mean response RTs 1015.2 msec). Condition effect also emerged [F(3,135) = 93.33; *p* < 0.001, *ηp*^2^ = 0.67], because all groups were slower in response RTs to target stimuli in the 4T and UN compared to 1T and RE conditions (all *p* < 0.001); no difference emerged between the 1T and RE conditions (*p* = 0.17). Finally, no significant interaction Group per Condition emerged [F (3,135) = 1.45; *p* = 0.23, *ηp*^2^ = 0.03].

### 3.2. Correlation Analysis

Results of the correlational analysis between the performance in the experimental paradigm and the global indexes of the CPRS-R were shown in Table 2. As concerns the BC, no significant relationship was shown between each CPRS index and the accuracy or the speed in this condition. Moreover, no relationship was found between the Global index Emotional lability and all experimental variables.

As concerns 1T, some relationships emerged for the ongoing task but not for the PM task. In particular, moderate negative relationships were found between the ongoing task accuracy and the ADHD index, the DSM inattentive, the DSM hyperactive/impulsive, and the DSM Total, i.e., the higher the CPRS score (more symptoms), the lower the accuracy in the ongoing task was (more impaired). An inverse relationship, but in the same direction, was found between the ongoing task response RTs and the score of some CPRS indexes. In particular, a moderate positive relationship emerged with the ADHD index, the Global index, and the DSM inattentive, i.e., the higher the score (more symptoms), the slower the responses were (more impaired).

As concerns 4T, negative moderate to strong relationships emerged between the ongoing task accuracy and all other CPRS indexes, with the exception of the Global index Emotional lability. In addition, a moderate positive relationship emerged between the ongoing task speed and the ADHD index, the Global index, the DSM Inattentive, and the DSM Total. In summary, the higher the CPRS score, the lower the performance in the ongoing task was. A similar relationship was detected in the PM task of the 4T condition, in which the higher the CPRS score in ADHD index, DSM Inattentive, DSM hyperactive/impulsive, and DSM Total, the lower the accuracy on the PM task was. At the same time, the higher the CPRS score in the DSM Inattentive and the DSM Total, the higher the response RTs were.

Elevation in ADHD index, DSM inattentive, and DSM Total negatively correlated with the accuracy in the UN (Ongoing and Prospective trials) and with the performance on the PM Task of the RE (lower accuracy and higher response RTs). Elevation in DSM Hyperactive/impulsive also negatively correlated with PM task accuracy of the UN and with the performance on the RE (lower accuracy on the ongoing and PM tasks, higher response RTs on the PM tasks). DSM Total positively correlated with the response RTs on the PM tasks of the UN. Finally, ADHD index and DSM Inattentive positively correlated with the ongoing task response RTs of the RE condition.

However, after applying Bonferroni correction for multiple comparisons, only the strong negative correlation between DSM Hyperactive/impulsive index and the ongoing task accuracy of the 4T condition survived (see Table 2).

## 4. Discussion

The current study examined event-based PM in children with ADHD compared to age-, gender- and IQ-matched control participants. We investigated the presence of a possible PM deficit in children with ADHD in terms of impairment in different PM tasks, as well as a possible interfering effect of the PM task on the ongoing task performance. We also evaluated the impact of three cognitive variables (WM load, attentional focus, and reward sensitivity) on event-based PM performance. Finally, we examined the correlation between event-based PM performance and ADHD symptoms (CPRS scores).

In the ADHD group, a deficit of event-based PM emerged in terms of impaired performance in both ongoing and PM tasks. Indeed, we documented the presence of a marked interfering effect in PM conditions for the ongoing task performance when the PM task was more demanding (4T and UN conditions), resulting in a weakening of response accuracy. We also detected higher reaction times in responding to PM targets for all the PM conditions and reduced accuracy under conditions requiring more cognitive resources (4T and UN). Finally, the correlation between PM performance and CPRS scores revealed a significant relationship between ADHD-related indexes and reduced accuracy/higher reaction times in the PM conditions (ongoing and PM trials), but not in BC. However, the strongest relationships emerged between the DSM Hyperactive/impulsive index and the accuracy on the ongoing task of the 4T (poorer performance associated with more severe symptoms), which is the WM demanding PM condition.

### 4.1. Event-Based PM Tasks Interfere with Ongoing Task Execution in Children with ADHD

Among children with ADHD, there was a significant tendency to have decreased ongoing task performances, resulting in a weakening of accuracy compared to their BC performance and compared to controls in the 4T and UN conditions. On the contrary, children in the CON group exhibited similar response accuracy for all conditions, thus suggesting that PM tasks did not interfere with their ongoing task performance. Conversely, analysis of the RTs did not detect any difference between groups. However, within-group comparisons revealed that children with ADHD were slower in executing the ongoing task in all PM conditions compared to BC. Conversely, again within-group analysis in the CON group failed to detect differences between response RTs in almost all PM conditions and BC. There was an exception for the UN, where participants were slower than all other conditions, likely because of the cost of the visual searching required to find the PM target. This finding is in line with previous research identifying cue centrality as a variable affecting children’s PM performance on typically developing children [52] and, more generally, it is consistent with literature indicating that spared event-based PM performance could be at the cost of ongoing task performance [23,53].

The cost to ongoing task performance in our ADHD group may reflect a described characteristic of individuals with ADHD, who show difficulty in adequately allocating cognitive resources in task performances [54]. A proposed model for ADHD deficit focuses on the imbalance between brain regions involved in higher-order cognitive control and the Default Mode Network, a set of brain regions active during “rest” or during less demanding tasks. The inability to adequately suppress the Default Mode Network while performing a task has been associated with attentional fluctuation and performance deficits in ADHD [55,56,57]. On the other side, the behavioral neuroenergetics theory of ADHD proposed that the slow processing speed is linked to an insufficient neuronal energy supply, leading to floating performance and diffusion of attention [58]. Thus, our results could be explained in light of these etiological considerations. PM tasks mostly arise in a complex and dynamic environment: children with ADHD perhaps exhibit a less accurate performance on the ongoing task of the 4T condition because of difficulties in distributing cognitive resources when the task is characterized by high levels of cognitive load. Moreover, children with ADHD exhibited a marked worsening of response accuracy on ongoing tasks when the PM target was located on the corner of the screen, outside the focus of attention. This finding is in line with previous literature demonstrating that ongoing task performance is negatively affected by PM tasks involving non-focal PM targets, thus suggesting some degree of capacity sharing between ongoing tasks and PM processing [9].

According to our preliminary hypotheses, WM load and attention focus seem to be crucial variables intervening in performances of children with ADHD, affecting ongoing task performance during event-based PM tasks to a greater extent than in controls, and having an impact on both accuracy and speed. These variables could therefore be identified as key components influencing event-based PM performance in individuals with ADHD.

### 4.2. Manipulation of the Reward Positively Influences Ongoing Task Performance in ADHD Children

Children with ADHD exhibited higher response accuracy on ongoing task performance of the RE condition compared to the other PM conditions. Intriguingly, differences in accuracy between the ADHD and CON groups at RE condition did not reach statistical significance. This suggests that the return to the BC performance level in the ADHD group could have been mediated by the manipulation of the reward amount. Of note, the analysis of response accuracy to PM targets revealed that the performance of the CON group was significantly lower in the UN than RE condition, thus confirming that the amount of reward also influences the performance of typically developing children. This is in line with previous findings linking memory function to emotional stimuli associated with the reward mechanism [59,60]. Therefore, consistent with our hypothesis, reward seems to play an important role in supporting the performance of children with ADHD at event-based PM tasks. Again, our results mirrored the clinical manifestation of ADHD. Indeed, it has been documented that individuals with ADHD exhibit high reward sensitivity, namely the reaction and sensitivity to reinforcement, including reward, punishment, and reinforcement schedules [61].

A possible explanation for such disruption in reward processing of individuals with ADHD could be found in the neurobiology of the disorder. Literature reported increased activity in reward regions–such as the ventral and dorsal striatum—in individuals with ADHD responding to reward delivery [62]; moreover, consistent findings indicated that individuals with ADHD have reduced ventral striatal activity in response to reward anticipation [63,64]. It has been proposed that this lower response to anticipated reward could be related to amplified reward-seeking behaviors, correlated with symptoms of hyperactivity/impulsivity, acting as compensation for the reduced ventral striatum activity [63]. Accordingly, individuals with ADHD exhibit the tendency to attribute great weight to immediate rewards and to be particularly prone to frustration in cases of delayed reward [40,64].

Overall, our findings confirm the already established role of reward in cognitive performance not only for children with ADHD but also for typically developing children. Literature documented that reinforcement positively affects the performance of children with ADHD in a stop-signal task [65], in a time reproduction task [66], and ameliorates response inhibition [67]. Our results extend this knowledge to PM performance, providing further insight into novel outcomes for behavioral treatments.

### 4.3. PM Performance Is Related to ADHD Symptoms

Interestingly, performance in the ongoing task per se was not related to any CPRS score, in the BC. However, the performance in the ongoing task was negatively associated with many ADHD-related symptoms when a PM condition was proposed to participants, thus indicating a modulatory effect of ADHD-related symptoms in the presence of PM tasks. Moreover, the performance on the PM conditions was never associated with the Global index-Emotional lability, which is not a core symptom of ADHD. The most consistent finding was the adverse relation between elevations in many ADHD-related symptoms, such as ADHD index, global index, DSM Inattentive, DSM Hyperactive/impulsive, DSM Total and performance (both accuracy and speed) in ongoing and PM trials of several PM conditions. However, the strongest negative association emerged between the DSM Hyperactive/impulsive index and the ongoing task accuracy on the 4T (WM demanding). Notably, the performance on the ongoing task in the 4T was one of the poorest in the group with ADHD. It could be hypothesized that the severity of ADHD symptoms interferes with resource allocation to the ongoing task, rather than to the PM task [11,31]. In particular, it can be speculated that ADHD symptoms are related to an overall difficulty in distributing attentional resources, which makes children with ADHD struggling in attending to both ongoing and PM tasks.

These results suggest that the slower reaction times and the lower accuracy in responding to PM targets or during the ongoing task in the PM conditions are associated with core features of ADHD. Thus, the event-based PM performance could be related to ADHD symptoms.

Although preliminary, these findings are quite promising because they pave the way to further examination of PM as a possible neuropsychological variable discriminating between individuals with ADHD and the general population. This is consistent with literature reporting an association between the severity of ADHD symptoms (e.g., hyperactivity) on CPRS and time-based PM difficulties [22]. Extending these findings to event-based PM might have a crucial clinical impact, considering that the diagnosis of ADHD is merely clinical since no objective confirmatory method is currently available. Indeed, the presence of specific behaviors in various contexts represents the most successful method of identifying ADHD and, in spite of the documented presence of structural and functional brain peculiarities [68] and an estimated high heritability of 74% [69], these elements are not diagnostically specific. Moreover, the neuropsychological assessments currently available for ADHD are of little use for diagnostic purposes: the most commonly used neuropsychological batteries and tests proved to be of limited utility for distinguishing individuals with ADHD from the general population, both in childhood [70] and in adulthood [71]. Our results pave the road for further investigations on the identification of an ADHD-specific neuropsychological marker supporting the diagnosis, which would contribute significantly to the identification of such a complex condition and to the setting-up of targeted interventions.

Certain limitations of this study must be underlined. First, the experimental task had limited ecological validity, which could, at least partially, reduce the extendibility of our results to real-life scenarios. Nevertheless, as previously discussed, our findings could mirror some clinical manifestations of ADHD, as also suggested by the documented correlation of the performance in our task with core features of ADHD. Another limitation is the lack of a comprehensive assessment of participants’ neurocognitive profiles, which could perhaps reveal the relative impact of important cognitive variables, such as WM abilities, on PM performance in children with ADHD. Thus, further investigations could help to understand the role of individual features in determining the performance at PM tasks in children with ADHD.

## 5. Conclusions

The current study provides important information about event-based PM functioning in children with ADHD. PM is linked to future orientation and planning, which are essential human features: deficits in PM performances have a number of consequences on daily-life skills and this might partially account for the phenotype of many individuals with ADHD. Thus, the possibility to assess the presence of PM deficits at an early stage represents a fundamental step toward prompt and adequate management of children with ADHD, according to their individual and specific needs.

The evidence for the role of WM load and attentional focus in PM performance of children with ADHD provides further insight into the cognitive system underlying PM impairment in ADHD. Moreover, this finding offers a basis for the setting up of efficient strategies to support children with ADHD in their daily-life activities. More specifically, the ascertainment of PM deficits in children with ADHD represents a crucial starting point for the setting up of tailored interventions. Presently, there is a range of neuropsychological approaches for PM rehabilitation in adults, such as compensatory and remediation strategies [72]. These forms of intervention grounds on two different approaches. The process-based approach targets specific cognitive abilities, such as WM, to improve PM accuracy [73,74]. On the other hand, the strategy-based approach aims to provide a mnemonic strategy to complete a PM task [74]. However, research on PM rehabilitation in pediatric age is currently limited, with some evidence of efficacy for other neurologic conditions such as traumatic brain injury [72,75]. To the best of our knowledge, specific rehabilitative approaches for PM in children with ADHD have not yet been tested. This could be explained, at least in part, by the fact that PM deficits in this population are considered to be related to impairment in executive functions, motivation abnormalities, and inattention [27,76,77,78]. It is conceivable then that already existing interventions, based, for instance, on motivational enhancement and the choice of manageable goals [79], might provide some indirect benefit for PM performance in children with ADHD, even though such aspects are not yet considered as outcome variables of available interventions. Further research is required to investigate the feasibility of the existent intervention to ameliorate PM performance in children with ADHD.

Another intriguing aspect to take into account for future research is linked to gender-related differences. Literature, in fact, suggests that task complexity influences response control in children with ADHD in a sexually dimorphic manner: in particular, among the population with ADHD, girls seem to be more prone than males to the interference effects deriving from task complexity [80]. Studies to extend our knowledge about gender-related effects on PM deficits would be an important contribution to understanding the pathophysiology of ADHD. 

Finally, our study has significant clinical implications: although we are far from the identification of a neuropsychological marker pathognomonic of ADHD, the correlation of PM deficit with CPRS scores may represent a tool to support clinical diagnostic efficiency of existing evaluation instruments.

## Figures and Tables

**Table 1:** Characteristics of participants.

Measure	ADHD N 24; 15M/9F		CON N 23; 16M/7F			
	M	SD	M	SD	F(1,45)	*p*
CA	10.42	(1.34)	10.15	(1.51)	0.44	0.51
IQ	108.12	(10.05)	112.39	(9.15)	2.31	0.14
CPRS Oppositional	63.35	(15.45)	46.37	(13.96)	13.70	<0.001 *
CPRS Cognitive probl./inattention	75.33	(12.72)	43.79	(5.75)	100.27	<0.0001 *
CPRS Hyperactivity	66.71	(14.40)	45.11	(8.36)	33.66	<0.0001 *
CPRS Anxious	54.09	(13.78)	45.79	(7.15)	5.62	0.02
CPRS Perfectionism	51.52	(11.41)	46.69	(10.70)	1.98	0.17
CPRS Social problems	57.87	(14.17)	46.95	(12.01)	7.20	0.01
CPRS Psychosomatic	58.65	(16.90)	47.68	(7.45)	6.87	0.01
CPRS ADHD index	75.00	(12.12)	46.47	(9.88)	68.90	<0.0001 *
CPRS Global index- Restless-impulsive	67.33	(11.75)	45.42	(8.91)	45.36	<0.0001 *
CPRS Global index-Emotional lability	60.04	(16.82)	48.26	(11.50)	6.71	0.01
CPRS Global index	69.67	(13.78)	45.68	(10.36)	39.68	<0.0001 *
CPRS DSM Inattentive	75.04	(14.44)	45.68	(8.10)	62.69	<0.0001 *
CPRS DSM Hyperactive/impulsive	64.79	(12.60)	46.63	(9.19)	27.73	<0.0001 *
CPRS DSM Total	73.46	(13.27)	44.37	(7.68)	72.00	<0.0001 *

ADHD = group with Attention deficit/hyperacticity disorder; CON = control group; CA = chronological age; IQ = intelligence quotient; CPRS = Conner’s Parent Rating Scales; * survived after Bonferroni correction.

**Table 2:** Correlational analysis between the results in the experimental paradigm and the global indexes of the Conner’s Parent Rating Scales in both the studied groups.

CPRS	BC		1T				4T				UN				RE			
			Ongoing		PM		Ongoing		PM		Ongoing		PM		Ongoing		PM	
	Acc	Speed	Acc	Speed	Acc	Speed	Acc	Speed	Acc	Speed	Acc	Speed	Acc	Speed	Acc	Speed	Acc	Speed
ADHD Index	−0.19	0.19	−0.38 *	0.38 *	−0.27	0.17	−0.40 **	0.36 *	−0.32 *	0.28	−0.35 *	0.12	−0.32 *	0.29	−0.21	0.31 *	−0.32 *	0.34 *
Global index- Restless-impulsive	−0.13	0.09	−0.26	0.30	−0.12	0.09	−0.30 *	0.26	−0.17	0.19	−0.24	0.17	−0.30	0.24	−0.19	0.23	−0.22	0.33 *
Global index-Emotional lability	−0.03	0.18	−0.14	0.14	−0.20	−0.03	−0.26	0.11	−0.13	0.04	−0.22	0.25	−0.17	0.18	−0.14	0.11	−0.07	0.23
Global index	−0.13	0.08	−0.24	0.31 *	−0.18	0.04	−0.34 *	0.33 *	−0.22	0.20	−0.25	0.21	−0.26	0.24	−0.19	0.24	−0.23	0.26
DSM Inattentive	−0.21	0.22	−0.37 *	0.40 **	−0.26	0.22	−0.40 **	0.39 *	−0.32 *	0.31 *	−0.37 *	0.11	−0.36 *	0.28	−0.23	0.32 *	−0.32 *	0.36 *
DSM Hyperactive/impulsive	−0.18	0.04	−0.37 *	0.16	−0.23	0.06	−0.52 **^^^	0.21	−0.32 *	0.26	−0.28	0.20	−0.35 *	0.30	−0.33 *	0.26	−0.36 *	0.43 **
DSM Total	−0.18	0.08	−0.34 *	0.28	−0.28	0.12	−0.48 **	0.35 *	−0.34 *	0.36 *	−0.40 **	0.13	−0.45 **	0.36 *	−0.30	0.30	−0.32 *	0.42 **

CPRS = Conner’s Parent Rating Scales; BC = baseline condition; 1T = One target condition; 4t = Four-target condition; UN = Unfocal condition; RE = Reward condition; * *p* < 0.05; ** *p* < 0.01; ^survived after Bonferroni correction.

## Data Availability

The data presented in this study are available on request from the corresponding author.
